# UTILIZING CARE PREFERENCE ASSESSMENT TO UNDERSTAND THE NEEDS OF OLDER ADULTS AT ADULT DAY SERVICES (ADS) IN TAIWAN

**DOI:** 10.1093/geroni/igad104.2511

**Published:** 2023-12-21

**Authors:** Chih-ling Liou

**Affiliations:** Kent State University, Kent, Ohio, United States

## Abstract

Understanding individuals’ preferences and honoring them throughout the care delivery process is essential for care services. When preferences are understood and honored, care recipients perceive a greater sense of autonomy, which improves their quality of life and emotional well-being. Adult Day Services (ADS), a fast-growing community service offers daytime programs for those with physical and/or cognitive impairments. In order to better understand ADS attendees’ care preferences and to help ADS staff to deliver person-centered care in Taiwan, this study adapted the Preferences for Everyday Living Inventory for ADS in Chinese (PELI-ADS-C) to assess preferences for activities and lifestyle experiences. Forty-three interviews were conducted with older adults recruited from three different ADS centers in Taiwan. The majority of participants were females (85%) with ages ranging from 67 to 93 years old. Among the 43 preference items, “do things with groups of people” and “participate in religious services or practices” were rated as most important followed by “reminisce about the past” and “exercise.” “Volunteer your time” and “gender of the staff taking care of you” were rated as least important. Participants shared that “It is not good for us to be alone.” “It is more fun for me to be with a group of people.” “I had no time to be a volunteer when I was younger. Now, I have to attend the center every day with no time to be a volunteer.” Results can be shared with ADS staff to develop care and activities that corresponded with attendees’ preferences.

